# 重视肺结核的外科治疗

**DOI:** 10.3779/j.issn.1009-3419.2018.04.20

**Published:** 2018-04-20

**Authors:** 言峥 宋

**Affiliations:** 上海市（复旦大学附属）公共卫生临床中心胸外科 Department of Thoracic Surgery, Public Health Clinic Center of Shanghai (Affiliated to Fudan University)

外科技术用于结核病的治疗已有一百余年的历史，时至今日，外科仍然是一种不能被替代的、和化疗相辅相成的结核病治疗手段。外科可以用引流术、清除术和切除术等方法将结核病变移出体外，用以减少患者身体内的结核分枝杆菌负荷，切除传染源，减少结核中毒症状，以使有效的结核化疗药物将术后残存的结核分枝杆菌杀灭，从而达到治愈的目的，这对结核病控制和个体治疗都是非常重要的。

## 1 外科手术与结核病控制

我国的肺结核外科治疗，一直将痰菌阳性的肺结核作为手术对象之一，彰显了外科在控制肺结核传染源的独特性。

1959年，吴英凯、邵令方等撰写的《新中国十年来肺结核外科疗法的成就》^[1]^文中，报告肺结核外科治疗病例6, 116例，术后的痰菌阴转率达91%，说明外科的干预在消灭肺结核传染源中的作用十分重要。

肺结核短程化疗的成功，使得大多肺结核可以通过单纯化疗获得成功。因此，1985年，国内制定了第一个“肺结核外科适应证草案”^[2, 3]^，草案中特别提出对空洞型肺结核、毁损肺、结核球、肺结核大咯血、脓胸、支气管胸膜瘘作为手术适应证等，并强调指出外科治疗的范畴应包括痰菌阳性或者长期阴性有明显症状者，也就是说专家们清醒地意识到外科在控制肺结核传染源的作用，1993年，中华结核和呼吸杂志编委会在无锡召开的肺结核肺癌外科手术适应证研讨会肺结核手术适应证标准（试行草案）^[4]^，同时专门发了述评，讨论当前肺结核病的内科治疗，并兼论不失时机地转外科治疗^[5]^，这个试行草案奠定了我国近20余年来肺结核外科治疗基础，草案依然把痰菌阳性，控制传染源作为主要目标，同时兼顾了患者的个体化治疗。草案认为：空洞性肺结核手术适应证是经抗结核的药物初治和复治规则治疗（约18个月）空洞无明显变化或增大，痰菌阳性者，特别是结核菌耐药的病例。肺结核瘤手术适应证为结核瘤经规则抗结核治疗18个月，痰菌阳性，咯血。毁损肺手术适应证为经规则抗结核治疗仍排菌或反复咯血及继发感染者。鉴于化疗期间或者化疗结束菌阴结核病的痰液仍有一定的毒力和传染性，所以肺结核外科适应证的制定总是将控制传染性放在第一位。遗憾的是，草案虽然提到了耐药结核菌病例应积极手术，但是没有出台细则，并且过多地强调了化疗的作用，把1985年的肺结核术前用药9个月改为18个月。2005年临床技术操作规范（结核病分册）^[6]^，重新整理了肺结核手术适应证，与1993年试行方案无大的改变。总之，从历史上看，外科在控制结核病传染源上的作用和地位是积极的，不可替代的。

随着耐药结核病人逐渐增加，特别是1992年，美国疾病控制中心（Centers for Disease Control and Prevention, CDC）正式提出“耐多药结核病”（multi-drug resistant-tuberculosis, MDR-TB）的概念后，世界卫生组织（World Health Organization, WHO）重新评估了全球耐药结核病的严峻疫情，并于2006年，WHO制定了“耐药结核病规划管理指南”^[7]^，并把外科治疗结核病作为重要的治疗方法之一，成为在控制结核病传染源积极的，与化疗手段平行的治疗手段。之后的2008版、2016版WHO耐药结核病规划管理指南^[8, 9]^都将外科作为一个积极的治疗手段，而不是作为辅助治疗手段治疗有手术指证的MDR-TB。指南中建议耐多药肺结核的手术适应证为：痰菌阳性，多耐药或耐多药，局限的病变。WHO倡议的耐多药肺结核的手术适应证自2005年至今标准未变。很多文献，包括*meta*分析也为WHO的耐多肺结核外科指南提供了依据。国内傅瑜、肖和平等在外科治疗耐多药肺结核综合治疗中的作用中认为，有外科参与的耐多药肺结核综合治疗模式可大大提高其治愈率。Xu^[10]^在4, 996篇肺结核文献中，分析了15篇耐多药肺结核外科手术文献，得出结论，外科手术的参与，使肺结核的治愈率可升高至84%，远高于耐多药肺结核平均50%的治愈率。

遗憾的是，国内尚没有耐多药肺结核的外科诊疗指南出台。

## 2 慎重选择肺结核手术时机和手术方法

钱元福^[11]^在1957年详细地介绍了肺结核的外科治疗的各种方法及手术适应证：①萎陷手术（膈神经麻痹术、胸膜粘连烙断术、人工气胸、胸膜外油胸、胸廓改形术、骨膜外塑胶球填塞术）；②肺切除术（全肺切除、肺叶切除、肺段切除、楔形切除）；③其他如空洞引流术、空洞切开术、肺叶外翻、肺动脉结扎术等。手术原则是对比较局限的不易自行闭合的空洞型肺桔核可多考虑肺切除术，对较广泛而高度纤维化的肺结核可考虑胸廓成形术等萎陷手术。这是肺结核外科治疗最为活跃的时期，手术技术多样且逐步成熟，但作者最后也指出手术方式需很根据患者的年龄、生活情况和主观愿望以及医院的设备等条件选择。这与宋言峥等^[12]^提倡的LTB-S结核外科手术时机和方法选择非常类似，即：根据患者本人体质和主管愿望、病变情况、抗痨时间等选择合适的手术方式。

但是，由于如果没有有效的抗结核药物保护及手术适应证选择、手术技巧等因素的影响，肺结核的手术后并发症如支气管胸膜瘘、术后脓胸形成等非常常见，文献报道15%-30%不等^[10]^。这期间，很多作者不断积极改善手术技巧，降低术后并发症发生率，如北京胸科医院^[13]^胸外科50年代-60年代国内率先开展的“结核支气管残端粘膜外缝合法”大大降低了支气管残端瘘的发生率。Iseman^[14]^对29例同时耐利福平和异烟肼耐患者的外科治疗效果的报道，结果显示25例转阴，27例存活。取得了很大的成功，其提倡的手术适应证：广泛耐药引起治疗失败和复发的可能性增加；影像学上局限的病变，并且有足够的心肺功能储备；有充分的药物减少结核分支杆菌负荷，以满足支气管切除残端的愈合。这些观点逐渐被接受，接着，很多文献报道对MDR-TB患者实施外科治疗^[15, 16]^，因病人选择的多样性（双侧、支气管膜结核、体重指数等）不同等，痰菌转阴率和术后并发症发生率不等，最高的痰菌阴转率达96.3%^[17]^。手术时间建议术前按耐药治疗方案抗痨治疗至少2个月^[17-20]^，同时建议手术由经验丰富的医生主刀。

## 3 支气管结核的外科治疗

支气管结核（支气管内膜结核）是肺结核的一种特殊类型，约10%-40%的活动性肺结核患者合并有支气管结核，此类疾病在临床上最易被漏诊，女性多见，单纯抗结核治疗时间长，部分病例达1年以上的治疗，特别是合并耐多药肺结核的病例，时间可长达24个月以上，且易遗留不同程度的并发症如支气管狭窄、支气管扩张等。介入治疗学的兴起如支气管镜下的冷冻治疗、球囊扩张等方法，使得支气管结核在药物治疗和外科治疗之间多了一种选择，并且大部分病人可从中获益。文献报道中合并支气管内膜结核的肺结核术后的支气管胸膜瘘发生率高，这不仅与术前的化疗时间及效果有关，也与手术切缘是否有结核病病变残留及是否包埋等残端处理方法相关。确保手术切缘无病变是最基本的手术方法和恰当的选择。本文作者对支气管结核的手术适应证的选择无疑是很慎重的，局限于一叶和一侧的结核病变，经术前充分有效抗痨及采用冷冻和球囊扩张等介入治疗效果不好的支气管狭窄和扩张的患者实施手术切除，术后无支气管胸膜瘘等严重并发症，继续抗结核治疗9个月-12个月，取得满意的效果。术后的化疗方案选择常根据术后手术标本中结核分枝杆菌的药敏和菌种鉴定的结果再进行调整。值得重视的是，易漏诊的支气管内膜结核术前常规支气管镜检查是必须的，手术适应证、手术方法和手术时机均依赖于支气管镜下表现。总之，综合治疗是支气管内膜结核治愈的保障，选择合适的患者进行恰当的外科干预，能取得较好的临床疗效。

## 4 目前外科诊疗结核病存在问题及展望

### 4.1 菌阴肺结核的外科治疗

目前肺结核的治愈标准之一是疗程结束时痰结核分枝杆菌阴性，但是阴性并不意味着治愈。Shiraishi等^[21]^报道，切除肺标本的细菌学检测表明，27%术前痰菌阴性的病人在目标病变中仍然隐匿着活的菌株，国内冯慧芝等^[22]^从化疗后手术切除的肺标本，发现术后组织切片可找到结核菌，其中组织结核菌培养阳性率可达35%，显示出这些患者必须采取外科干预的方式将其移出体外个体方能治愈，痰菌阴性的患者可能隐藏着极大的复发风险，进而成为新的传染源。

### 4.2 耐药肺结核的外科干预率

对耐多药肺结核病人，和国外相比，国内对耐多药肺结核的外科干预率要比国外低1倍以上^[23, 24]^，而国内的耐多药肺结核外科手术指南还没有出台。

### 4.3 结核病术后快速诊断和外科诊断研究被忽视

“从病灶中来，再到病灶中去”，外科在处理结核病病灶方面具有得天独厚的优势，如何在这些手术标本上做些深入的有指导患者化疗和手术的研究，非常有意义。冯惠芝等^[22]^从结核瘤的组织细菌学检查探讨结核瘤手术适应证，给了我们很多启示。我们认为，控制结核病需要发现结核病新的TB标志物进行诊断，这些标志物的发现大都需要从结核病的病灶标本中来获取。如果能够发现判断早期或者预测肺结核患者化疗可能失败的指标，对局限的病例及早进行手术干预，就可以降低发病率。

分子生物学方法为结核病的术后快速诊断提供了技术支持，术后24 h手术组织标本的分子生物学检测如耐药检测，菌种鉴定等技术，能使内科专家们在术后今早制定合适的化疗方案，巩固手术疗效，减少并发症和复发率。
